# Low-bias photoelectrochemical water splitting via mediating trap states and small polaron hopping

**DOI:** 10.1038/s41467-022-33905-6

**Published:** 2022-10-20

**Authors:** Hao Wu, Lei Zhang, Aijun Du, Rowshanak Irani, Roel van de Krol, Fatwa F. Abdi, Yun Hau Ng

**Affiliations:** 1 Low-Carbon and Climate Impact Research Centre, School of Energy and Environment, City University of Hong Kong, 83 Tat Chee Avenue, Kowloon, Hong Kong SAR China; 2 City University of Hong Kong Shenzhen Research Institute, Shenzhen Hi-Tech Industrial Park, Nanshan District, Shenzhen, China; 3 School of Chemistry and Physics and Centre for Materials Science, Faculty of Science, Queensland University of Technology, Gardens Point Campus, Brisbane, QLD 4001 Australia; 4 Institute for Solar Fuels, Helmholtz-Zentrum Berlin für Materialien und Energie GmbH, Hahn-Meitner-Platz 1, Berlin, 14109 Germany

**Keywords:** Photocatalysis, Photocatalysis, Nanoscale materials, Solar fuels

## Abstract

Metal oxides are promising for photoelectrochemical (PEC) water splitting due to their robustness and low cost. However, poor charge carrier transport impedes their activity, particularly at low-bias voltage. Here we demonstrate the unusual effectiveness of phosphorus doping into bismuth vanadate (BiVO_4_) photoanode for efficient low-bias PEC water splitting. The resulting BiVO_4_ photoanode shows a separation efficiency of 80% and 99% at potentials as low as 0.6 and 1.0 V_RHE_, respectively. Theoretical simulation and experimental analysis collectively verify that the record performance originates from the unique phosphorus-doped BiVO_4_ configuration with concurrently mediated carrier density, trap states, and small polaron hopping. With NiFeO_x_ cocatalyst, the BiVO_4_ photoanode achieves an applied bias photon-to-current efficiency of 2.21% at 0.6 V_RHE_. The mechanistic understanding of the enhancement of BiVO_4_ properties provides key insights in trap state passivation and polaron hopping for most photoactive metal oxides.

## Abstract (web summary)

While photoelectrochemical water splitting produces fuel from solar energy, a large fraction of photoanode photoexcited charge carriers cannot be extracted efficiently at low bias voltages. Here, authors improve the charge transport in P-doped BiVO_4_ by mediating polaron hopping and trap states.

## Introduction

Hydrogen production via photoelectrochemical (PEC) water splitting is an underpinning technology to converting intermittent solar energy into storable and transportable chemical fuels^1^. Developing efficient photoelectrode materials has proved to be highly demanding, especially for photoanodes, due to sluggish water oxidation reaction kinetics and anodic (photo)corrosion^2^. Monoclinic bismuth vanadate (BiVO_4_) is particularly interesting; it was first used as a powder photocatalyst in suspension system^3^ and further developed to be a top-performing metal oxide photoanode to photoelectrochemically split water^4^. However, its poor charge carrier transport leads to severe bulk and surface charge recombination, preventing BiVO_4_ photoanodes from achieving the theoretical maximum activity^5^, particularly at low-bias voltage. The poor charge transport is mainly due to trap-limited and polaron-bounded electron transport^6,7^.

Most studies of modified BiVO_4_ photoanodes achieved relatively high performance only with the assistance of relatively high bias voltages, typically up to 1.23 V vs. reversible hydrogen electrode potential (V_RHE_)^8–12^. When BiVO_4_ photoanodes operate at lower bias voltages, a large fraction of the photoexcited charge carriers cannot be extracted efficiently, resulting in recombination losses by potential “traps” along the transport pathway. As a result, the reported charge separation efficiencies at potentials ≤0.6 V_RHE_ remain lower than 70%^13–16^.

Recent studies have uncovered that native defects^17,18^ may introduce deep and/or mid-bandgap trap states, stimulating photoinduced carrier trapping and nonradiative recombination^19^. However, even when intrinsic defects have been alleviated, the low electron mobility and poor charge carrier transport are not fully overcome. This has recently been attributed to small polaron formation^20–23^, which ultimately determines the excited state carrier dynamics of BiVO_4_. The movement of small polarons occurs via hopping to overcome the energy barriers induced by the local polarization of the lattice and is much slower than band transport. Like deep or mid-bandgap trap states, small polaron formation can undermine the performance of BiVO_4_ mainly in two ways, i.e., by limiting the photoinduced Fermi-level splitting (photovoltage)^24,25^ and by increasing the recombination losses^26^.

Herein, we combine climbing nudged elastic band (cNEB) simulation and temperature-dependent conductance measurements and provide evidence for the decrease of polaron hopping activation barriers of BiVO_4_ photoanodes upon incorporating phosphorus. Transient open circuit potential (OCP) measurements confirm that phosphorus doping also passivates the shallow and deep trap states that are intrinsically formed on the BiVO_4_ surface, thereby increasing the open-circuit photovoltage. Time-resolved microwave conductivity measurements (TRMC) reveal that the phosphorus-doped BiVO_4_ photoanode has ~1.7 times longer lifetime, ~2.8 times higher mobility, and ~2.2 times longer effective diffusion length of charge carriers compared to the pristine sample. The modulated charge transport in phosphorus-doped BiVO_4_ by concurrently mediating the carrier density, polaron hopping, and trap state introduces efficient low-bias PEC water splitting.

## Results

### Characterization of phosphorus-doped BiVO_4_ samples

The synthesis recipe for BiVO_4_ thin films was adopted from the literature^4^. Subsequently, phosphorus doping in BiVO_4_ was achieved by exposure to locally generated phosphine gas and water vapor in a tube furnace (Supplementary Fig. 1). This approach enables precise control over the doping degree of the samples (details in Methods), producing three samples labeled P-BiVO_4_−0.5 mg, P-BiVO_4_−1 mg, and P-BiVO_4_−5 mg based on the phosphorus precursor amount of 0.5 mg, 1 mg, and 5 mg, respectively.

**Fig. 1:**
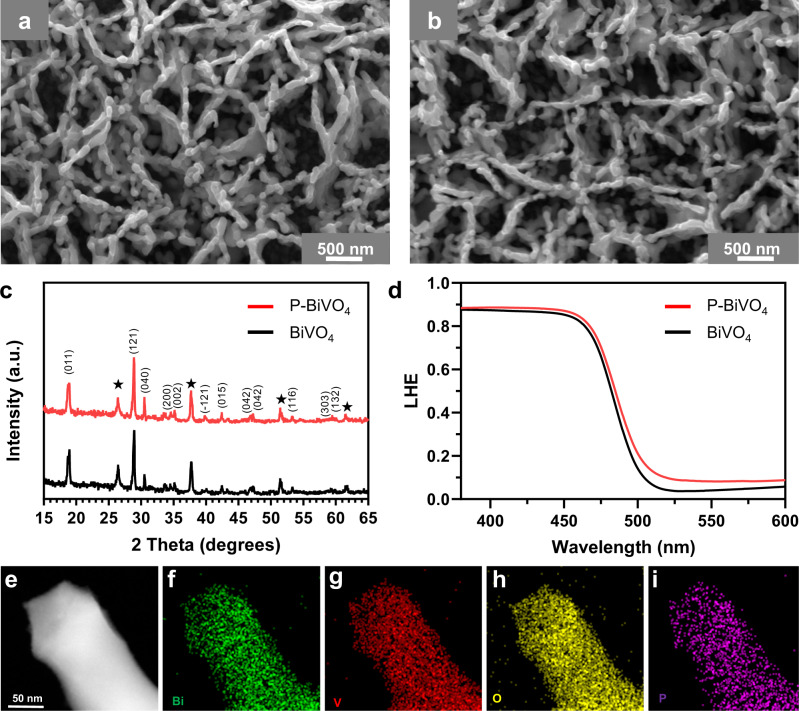
Morphological and optical characterizations. Scanning electron microscopy (SEM) image of **a** the pristine BiVO_4_ and **b** the phosphorus-doped BiVO_4_ sample (P-BiVO_4_-1 mg). **c** X-ray diffraction patterns and **d** light harvesting efficiency (LHE) derived from the UV-vis absorption spectra of the BiVO_4_ and phosphorus-doped BiVO_4_ samples. The asterisk (*) remark in **c** denotes the diffraction peak of the FTO substrate. **e** High-angle annular dark-field (HAADF) image and the corresponding energy-dispersive X-ray (EDX) mapping images of **f** Bi, **g** V, **h** O, and **i** P of the phosphorus-doped BiVO_4_ photoanode.

**Fig. 2:**
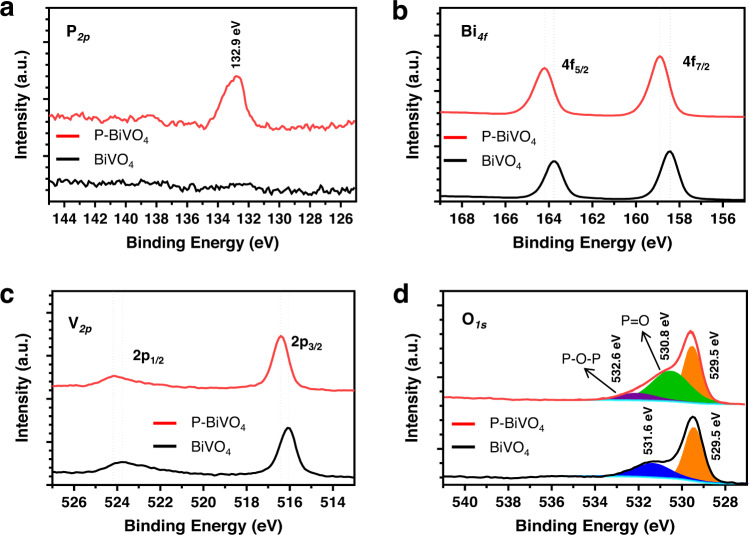
Electronic structure characterizations. **a** P 2p, **b** Bi 4 f, **c** V 2p, and **d** O 1 s XPS spectra of the BiVO_4_ and phosphorus-doped BiVO_4_ samples (P-BiVO_4_-1mg).

The scanning electron microscopy (SEM) images and X-ray diffraction (XRD) patterns of phosphorus-doped BiVO_4_ samples show negligible variations compared to the pristine sample (Fig. 1a–c and Supplementary Fig. 2a). A more detailed discussion regarding their morphological and crystallographic properties is provided in Supplementary Information. The high-angle annular dark-field (HAADF) image of the phosphorus-doped BiVO_4_ sample is shown in Fig. 1e. The energy-dispersive X-ray spectroscopy (HAADF-EDX) elemental maps illustrate that P, Bi, V, and O are homogeneously distributed within the phosphorus-doped BiVO_4_ sample (Fig. 1f–i), indicating that the phosphorus has been doped into the BiVO_4_ lattice without inducing segregated phase. The incorporation of phosphorus into the BiVO_4_ lattice is further confirmed from the X-ray photoelectron spectra (XPS), as shown in Fig. 2a–d and Supplementary Fig. 3a–d. The emergence of the P 2*p* peak indicates the presence of phosphorus in the BiVO_4_ lattice. The location of the P 2*p* peak (132.9 eV) agrees well with the P^5+^ ions in PO_4_^3-^ anions^27^, suggesting that the phosphorus ions reside at the V sites in the BiVO_4_ lattice. The Bi 4 *f* and V 2*p* peaks of phosphorus-doped BiVO_4_ samples are slightly shifted to higher binding energies than those of the pristine BiVO_4_. The shifts indicate a change in the local coordination of Bi and V ions. In previous studies on N-doped BiVO_4_, a shift to lower binding energies was observed and attributed to an increase in the concentration of oxygen vacancies^13,28^. Based on this, we tentatively assign the shift to higher binding energies after phosphorus-doping to a decrease in the concentration of oxygen vacancies. Note that oxygen vacancies are ubiquitous in the BiVO_4_ synthesized by the adopted strategy and in commercial samples^17,19^.

The O 1 *s* spectra of phosphorus-doped BiVO_4_ samples can be fitted using three main peaks (Fig. 2d). The lowest binding energy peak (529.5 eV) is assigned to the lattice oxygen of BiVO_4_. The other peaks at binding energies of 530.8 eV and 532.6 eV correspond to oxygen atoms in the P=O bond and the P-O-P bond of PO_4_^3-^ anions, respectively^27^. Indeed, with an increasing amount of phosphorus precursor, the ratio of the peak area between that of oxygen atoms in the P=O bond and the lattice oxygen of BiVO_4_ gradually increases, suggesting a reasonable control of the doping degree enabled by the gas-phase doping method (Supplementary Fig. 3d)^27^. The intensity of the peak at 531.6 eV, which is also present in the pristine BiVO_4_ and has been frequently assigned to surface-bounded hydroxyl radicals due to the oxygen vacancies of BiVO_4_^29^, is reduced.

The wavelength-dependent light-harvesting efficiencies (LHEs) were calculated from the measured transmission (T) and reflection (R) as LHE$=$1-T-R. The phosphorus-doped BiVO_4_ samples show a slightly shifted band-to-band absorption onset to longer wavelengths than the pristine sample (515 nm), as shown in Fig. 1d. Moreover, the phosphorus-doped samples show a stronger light absorption in the 380–600 nm wavelength range (Supplementary Fig. 2b). The improvement of the LHE at longer wavelengths is tentatively ascribed to phosphorus dopants with strong electron-donating ability, which modifies the atomic arrangement of BiVO_4_ with less strongly localized electrons and increases free-electron absorption.

### Photon-to-current conversion efficiencies

Initial photoelectrochemical measurements of the phosphorus-doped and pristine BiVO_4_ samples in a 0.1 M phosphate buffer (KPi) solution were done in the presence of a hole scavenger (SO_3_^2-^). The amount of precursor used for phosphorus-doping was optimized based on the incident photon-to-current conversion efficiency (IPCE) and photocurrent density results (Supplementary Fig. 4a, b), and the optimum amount of 1 mg was chosen for further PEC sulfite oxidation studies.

**Fig. 3:**
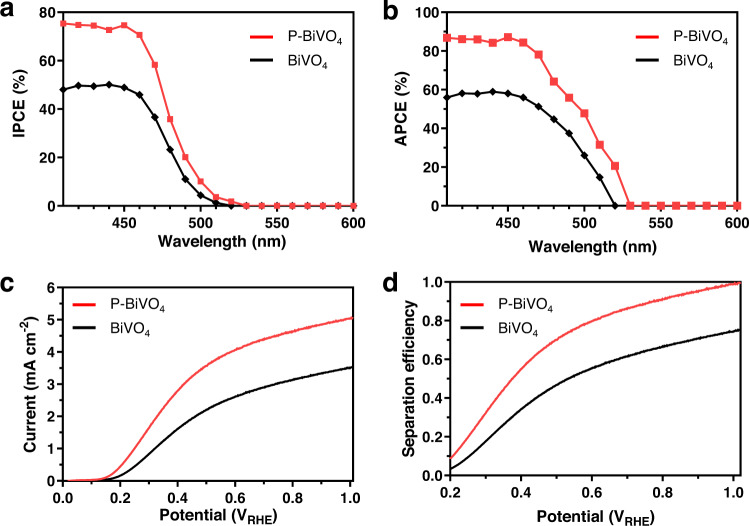
Photoelectrochemical performance for sulfite oxidation. **a** IPCE curves and **b** APCE curves of the BiVO_4_ and phosphorus-doped BiVO_4_ samples measured at 0.6 V_RHE_. **c** Current density-voltage curves of the BiVO_4_ and phosphorus-doped BiVO_4_ samples measured under rear side illumination with AM 1.5 G simulated light. **d** η_sep_ calculated from the current density-voltage curves for the BiVO_4_ and phosphorus-doped BiVO_4_ samples.

In Fig. 3a, the optimized phosphorus-doped BiVO_4_ sample shows enhanced IPCE values under monochromatic irradiance measured at 0.6 V_RHE_, and the IPCE onset is red-shifted by ~10 nm. The phosphorus-doped BiVO_4_ sample shows an IPCE value of 75% at 410 nm and an onset at ~530 nm. In comparison, the IPCE of the pristine BiVO_4_ is only 48% at 410 nm, and the onset is between 510 and 520 nm, which matches the measured LHE and the literature^13^. As the LHE of BiVO_4_ is slightly changed upon phosphorus doping, the absorbed photon-to-current conversion efficiency (APCE) offers a more precise evaluation of the internal photon-to-current conversion efficiency by BiVO_4_ samples. The calculated APCE values at 0.6 V_RHE_ of the phosphorus-doped BiVO_4_ are higher than those of the pristine sample (e.g., 87% vs. 56% at 410 nm, see Fig. 3b). The enhanced APCE indicates that the charge separation in BiVO_4_ was also improved in addition to the increased light absorption by incorporating phosphorus^4^.

As shown in the current density-voltage (*J-V*) plots measured under rear side illumination with AM 1.5 G simulated sunlight (Fig. 3c), the phosphorus-doped BiVO_4_ sample exhibits a photocurrent density for sulfite oxidation (*J*_sulfite_) of 4.08 ($\pm$0.2) mA cm^−2^ at 0.6 V_RHE_. In contrast, the pristine BiVO_4_ sample only produces a *J*_sulfite_ of 2.62 ($\pm$0.3) mA cm^−2^ under identical conditions. The phosphorus-doped BiVO_4_ sample shows a slightly negative shift of the onset potential (~50 mV) and a faster increase of photocurrent (i.e., higher slope) between 0.2 to 0.4 V_RHE_ compared with the pristine BiVO_4_. This indicates an improved fill factor, which likely arose from the work function tuning upon phosphorus incorporation^14^.

Integrating the product of the LHE of the phosphorus-doped and pristine BiVO_4_ samples with the solar photon flux gives the theoretical maximum absorbed photon-to-current density (*J*_abs_) of 5.10 mA cm^−2^ and 4.68 mA cm^−2^, respectively^14^. The charge separation efficiency (*η*_sep_) can then be calculated using the following relationship: *η*_sep_ $\approx$ *J*_sulfite_/*J*_abs_. The *J*-*V* curves measured at the potential between 0 to 1.23 V_RHE_ for the phosphorus-doped BiVO_4_ photoanode show the steeper rise of current densities above 1.0 V_RHE_ both under light irradiation and in the dark (Supplementary Fig. 5a), which indicates the arising of the electrochemical oxidation process of SO_3_^2−^. To avoid the contribution of current density from electrochemical SO_3_^2−^ oxidation, *η*_sep_ was calculated at the applied potential up to 1.0 V_RHE_. The *η*_sep_ of the phosphorus-doped BiVO_4_ measured at 0.6 V_RHE_ is 80%, which is ~1.43 times higher than that of the pristine sample (Fig. 3d). Remarkably, the doped BiVO_4_ achieves a record high *η*_sep_ of 99% at 1.0 V_RHE_^30–32^. The *η*_sep_ calculated from the net photocurrent density between 0 to 1.23 V_RHE_ for the phosphorus-doped BiVO_4_ photoanode was also provided (Supplementary Fig. 5b). We note that the excellent sulfite oxidation performances of the phosphorus-doped BiVO_4_ samples at low applied potentials (≤1.0 V_RHE_) are not only better than those of the state-of-the-art BiVO_4_ photoanodes (Supplementary Table 1) but also superior to those of other metal oxide photoanodes to date^33,34^.

### Insights into the charge transport mechanism

**Fig. 4:**
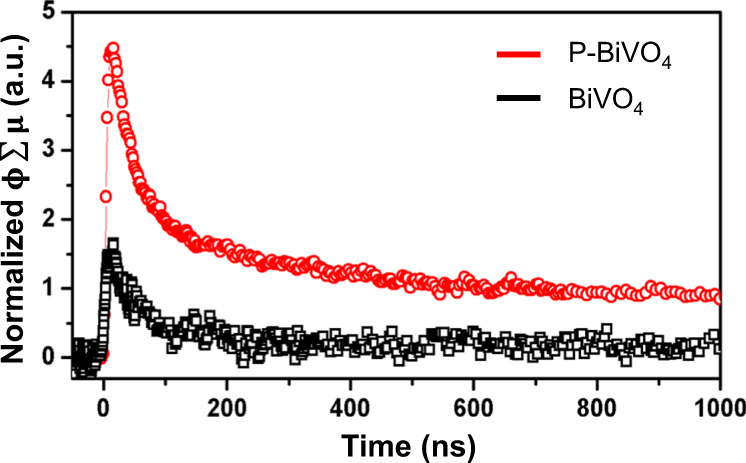
Charge transport dynamics of the BiVO_4_ and phosphorus-doped BiVO_4_ samples. Time-resolved microwave conductivity (TRMC) results measured under a pulsed laser light at 470 nm with an intensity of 2.5 × 10^14^ photons pulse^−^^1^ cm^−2^.

TRMC measurements further evaluated the charge carrier transport properties of the prepared samples. We are interested in how phosphorus incorporation affects the carrier mobility, lifetime, and diffusion length of BiVO_4_. Figure 4 shows the TRMC signals as a function of time for the pristine and phosphorus-doped BiVO_4_, measured under pulsed laser excitation of 470 nm with an intensity of 2.5 × 10^14^ photons cm^−2^ pulse^−1^. Based on the peak amplitude of TRMC signals ($\phi \sum {{{{{\rm{\mu }}}}}}$), the carrier mobility of BiVO_4_ increases by ~2.8 times upon phosphorus doping. The carrier lifetime, obtained from the exponential fit of the $\phi \sum {{{{{\rm{\mu }}}}}}$ signal transients, increases from 47.5 to 80.6 ns upon phosphorus doping. We also found that the effective diffusion length of the BiVO_4_ sample increases by ~2.2 times after phosphorus doping (details in Methods). TRMC measurements at other wavelengths of 410 nm and 450 nm were also performed (Supplementary Fig. 6a, b), showing comparable mobility increments of ~2.0 and ~2.5 times.

**Fig. 5:**
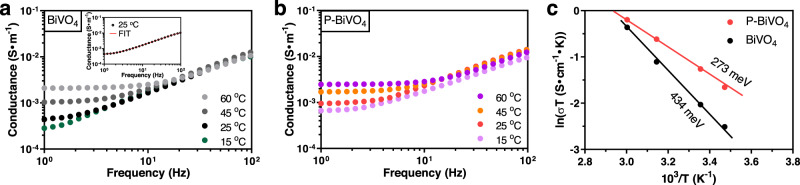
Temperature-dependent conductance of the BiVO_4_ and phosphorus-doped BiVO_4_ samples. Real part of conductance plots measured at different temperatures in the dark with a bias voltage of 0.6V_RHE_ for **a** pristine BiVO_4_ and **b** phosphorus-doped BiVO_4_ in the electrolyte of 0.1 M KPi buffer solution containing Na_2_SO_3_. Inset: fitted ac conductivity plot at 25^o^C for the BiVO_4_ sample. **c** DC Conductance fitting based on the small polaron transport derived from temperature-dependent conductance measured at 0.6 V_RHE_.

Recent reports suggested that the modest mobility of BiVO_4_ is due to a small polaron hopping mechanism^20–23^. The small polarons, which are electrons that are self-trapped in a local lattice distortion induced by the electron’s own charge, require thermal vibration energy to hop from one site to the next. The energy required for this process is known as the polaron hopping activation energy (*E*_a_). Specifically, the *E*_a_ of BiVO_4_ samples can be derived from the equation $\sigma (T)=A{T}^{-1}{\exp }\left(-{E}_{a}/{kT}\right)$, where *σ(T)* is the conductance at a particular temperature (*T*), *A* is a constant, and *k* is the Boltzmann constant. We determined the conductivity of BiVO_4_ electrodes at different temperatures (15 ^o^C–60 ^o^C) from dark EIS analysis with an applied bias of 0.6 V_RHE_. The real part of conductivity (${\sigma }^{{\prime} }\left(\omega \right)$) as a function of frequency (ω) for both BiVO_4_ electrodes with and without phosphorous doping at different temperatures is shown in Figs. 5a and 5b, respectively. The d.c. conductivity (${\sigma }_{d.c.}$) was determined by fitting the (${\sigma }^{{\prime} }\left(\omega \right)$ to the relation ${\sigma }^{{\prime} }\left(\omega \right)={\sigma }_{d.c.}+A\left(T\right){\omega }^{n}$, where the ${A}\left(T\right)$ is the temperature dependent frequency pre-exponential factor; and the exponent, n, generally varies between 0 and 1^35,36^. There is close agreement between the experimental and fitted data, as shown in the inset of Fig. 5a, typically for the BiVO_4_ sample at 25 ^o^C. Their conductivities were increased with raising the temperature, and the n values ranged from 0.6 to 0.8, suggesting the small polaron transport behavior^37,38^. Moreover, as further determined from the ${ln}\left[\sigma \left(T\right)\times T\right]-1/T$ plots (Fig. 5c), the *E*_a_ of BiVO_4_ decreases from 434 meV to 273 meV after incorporating phosphorus. It is worthy note that the experimental value of *E*_a_ for pristine BiVO_4_ is very close to the reported value of a recent work by Tang et al.^39^ The smaller *E*_a_ in the phosphorus-doped BiVO_4_ means that electrons require less energy to hop from one BiVO_4_ lattice site to the next, which likely arises from the changes of coordination environment induced by incorporating the phosphorus dopant. Such temperature-dependent conductance can also be attributed to charge transfer processes at the surface; however, this is unlikely be the only contributing factor considering the relatively high value of the activation energy that a facile hole scavenger was used in the electrolyte. The decrease of *E*_a_ also agrees well with the higher carrier mobility measured using TRMC. Further investigation on the variation of small polaron hopping behavior upon phosphorus doping is uncovered by computational studies, which will be discussed in the following section of theoretical calculations.

After investigating the charge transport, we are now in a position to understand the surface charge collection behavior of BiVO_4_ upon phosphorus doping. Previous studies have suggested that measuring the *N*_D_ is an effective method to compare the variations of depletion width (*W*_D_), thus investigating the band bending for hole collection at the semiconductor/electrolyte interface (SEI)^33^. As observed from the Mott-Schottky results (Supplementary Fig. 7), the phosphorus-doped BiVO_4_ has a positive shift of *E*_FB_ by 0.05 V and an increase of *N*_D_ by 1.5 times. We thereby found that the *W*_D_ of the BiVO_4_ sample decreases more than 18% upon phosphorus doping (details in Methods), suggesting a steeper band bending formed at SEI. The steeper band bending might enhance hole extraction and suppress surface recombination in the depletion region.

Moreover, the OCP profiles show that the phosphorus-doped BiVO_4_ has a higher photovoltage (V_ph_, 0.49 V) relative to the pristine BiVO_4_ sample (0.37 V) suggesting a more favorable thermodynamic driving force for the surface redox reaction (Supplementary Fig. 8). The pristine BiVO_4_ shows a more positive OCP_dark_ (0.48 V_RHE_) and a moderate OCP_light_ (0.11 V_RHE_), which is possibly due to Fermi-level pinning by surface states (trapping electrons at V^4+^ sites)^40^. On the other hand, the phosphorus-doped sample achieved an enlarged V_ph_ with an OCP_dark_ of 0.55 V_RHE_ and an OCP_light_ of 0.06 V_RHE_, attributed to the passivation of the surface states and the absence of Fermi-level pinning^14^. The resulting higher V_ph_ upon phosphorus doping is in good agreement with the negatively shifted onset potential shown in the *J–V* curves (Fig. 2c). The OCP decay transients further support the passivation of surface states in the BiVO_4_ sample upon phosphorus doping. Upon turning off the illumination, the transient OCP profile of the phosphorus-doped sample shows a faster decay than that of the pristine BiVO_4_. This suggests that the trap-limited recombination process at the surface states is mediated after incorporating phosphorus. More interestingly, the OCP response of the pristine BiVO_4_ is less reversible than that of the phosphorus-doped BiVO_4_, which indicates that more charge carriers are trapped in deep trap states of the pristine BiVO_4_ relative to the phosphorus-doped sample, likely at the BiVO_4_ surface^41^.

### Theoretical calculations

To offer insights into the superior charge separation performance of phosphorus-doped BiVO_4_ sample, the hopping processes of small polaron in BiVO_4_ with and without phosphorus doping were compared using the cNEB method. The position of phosphorus atoms in the lattice with charged states was again confirmed by theoretical calculations (details in Methods). Under the O-poor condition (Supplementary Fig. 9a), the interstitial phosphorus with a charge state of +5 can be the most stable defect only when the Fermi energy is lower than 0.33 eV, while in a wider range of Fermi energy from 0.33 to 2.1 eV, the substitution of V by phosphorus still possesses the lowest formation energy among the other possibilities (i.e., interstitial, Bi-substitution, and O-substitution). Under the O-rich condition (Supplementary Fig. 9b), the substitution of V by phosphorus is the most stable state for the whole range of Fermi energy within the bandgap of BiVO_4_. The results suggest that phosphorus likely replaces V and sits at its lattice sites in BiVO_4_, consistent with the experimental XPS observations.

**Fig. 6:**
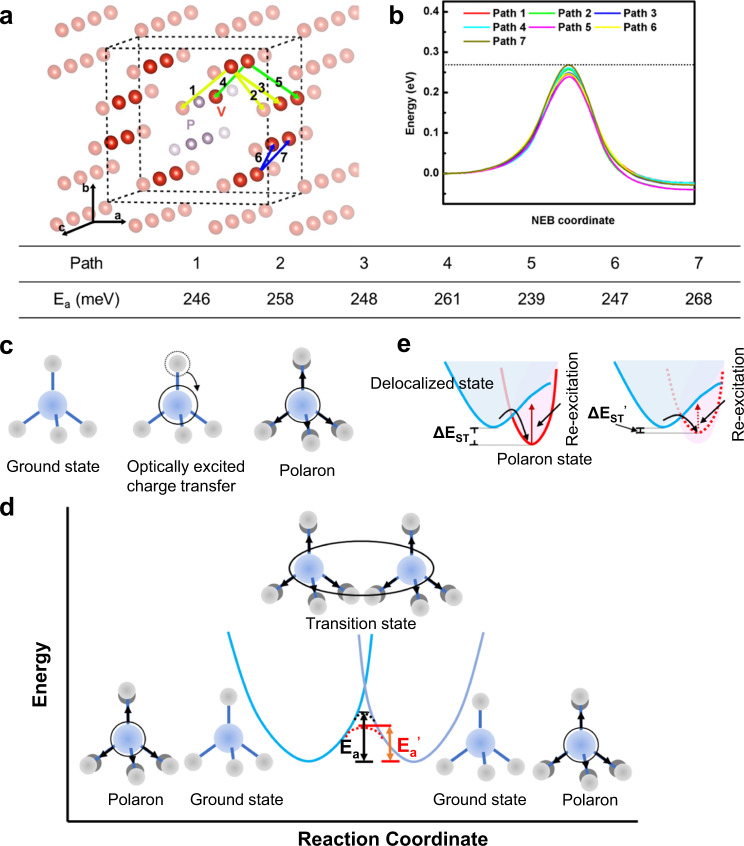
Small polaron hopping. **a** Schematic small polaron hopping paths in the phosphorus-doped BiVO_4_ sample for DFT calculations. Only V (red) and phosphorus (purple) atoms are displayed in the schematic structure model. Atoms highlighted are within the supercell and the rest are in the neighbor cells. The colored arrows are the selected hopping paths in the calculations. **b** Hopping energy barriers in phosphorus-doped BiVO_4_ sample along the reaction coordination of different reactions paths. The dashed line represents the hopping barrier in pristine BiVO_4_. The below table shows the hopping barriers of different paths in phosphorus-doped BiVO_4_. **c** Schematic small polaron formation: Initial excitation of BiVO_4_ leads to a charge transfer transition from oxygen (light grey) to V atom (blue). The initial excited carriers undergo optical-phonon scattering, during which the charge can be trapped in a small polaron, resulting the lattice expansion. Adapted and reprinted with permission from Rettie, A. J. E., Chemelewski, W. D., Emin, D. & Mullins, C. B. Unravelling small-polaron transport in metal oxide photoelectrodes. J. Phys. Chem. Lett. 7, 471–479 (2016). Copyright 2016 American Chemical Society. **d** Schematic small polaron hopping between two identical V atoms in VO_4_ units with potential energy landscape. Solid circles indicate the polaron location. The dark and red dashed curves indicate adiabatic surfaces of BiVO_4_ and phosphorus-doped BiVO_4_ samples, respectively. E_a_ and E_a_^’^ represents the polaron hopping energy barriers of BiVO_4_ and phosphorus doped BiVO_4_ samples. Adapted and reprinted with permission from Rettie, A. J. E., Chemelewski, W. D., Emin, D. & Mullins, C. B. Unravelling small-polaron transport in metal oxide photoelectrodes. J. Phys. Chem. Lett. 7, 471–479 (2016). Copyright 2016 American Chemical Society. **e** The potential energy surface for the ground and the photon excited states with polaron formation states and the corresponding re-excitation energy barriers (E_ST_) for BiVO_4_ (solid red) and phosphorus-doped BiVO_4_ (dashed red) samples.

The pristine BiVO_4_ sample was modeled by a $2\times 1\times 2$ supercell, while 3 V atoms were replaced with phosphorus atoms to simulate the doped sample according to the P to P+V ratio of 18% observed from XPS. Several substitution configurations were tested, and the one with the lowest energy was adopted (Fig. 6a). An extra electron was added to the simulated cell, which distorts one of the V atoms to form a small polaron. In pristine BiVO_4_, the polaron hopping in all directions is identical, and the barrier was calculated to be 268 meV (the dashed line shown in Fig. 6b). However, in phosphorus-doped BiVO_4_, the existence of phosphorus atoms breaks the symmetry of BiVO_4_, and the hopping path becomes anisotropic. Seven hopping paths between two adjacent V sites along different orientations were selected to investigate the polaron migration barriers in phosphorus-doped BiVO_4_ (Fig. 6a). The results show that the hopping barriers for most selected paths are smaller than that in pristine BiVO_4_, with the lowest barrier of 239 meV for the hopping path 5 (Fig. 6b and the inset table). As conductance is proportional to charge carrier mobility, the same exponential dependence on the migration barrier is expected: $\mu \propto {\exp }\left({-E}_{a}/{kT}\right)$. Based on this relationship, a decrease of *E*_a_ by 29 meV (path 5) results in a ~3.1 times increase of charge carrier mobility at room temperature, which is in a similar magnitude with the carrier mobility increment observed by TRMC. These results suggest that phosphorus dopants, with their strong electron-donating ability, are electrostatically repulsive to the small polaron V^4+^O_4_ unit^42^, offering a lower barrier to hop away from the adjacent V sites (Fig. 6c, d)^26,43^. Moreover, due to the stronger interactions between phosphorus and O atom, the simulated P-O bond distance is 1.559 Å, which is 14.3% shorter than that of the V-O bond with a small polaron (1.820 Å), creating a structural distortion in the lattice of BiVO_4_. Such structural distortion has also been reported to accelerate polaron migration^23,43^.

We note that the calculated results of *E*_a_ are relatively smaller than the experimentally measured values of pristine BiVO_4_^22,23,38^. We attribute the difference to the difficulty in experimentally quantifying the intrinsic oxygen vacancies in BiVO_4_. Recent studies have suggested that excessive oxygen vacancies could coulombically hinder small polaron migration^13,17^. Since oxygen vacancies were not quantified and considered in our modeling, the smaller simulated values relative to experimental data are expected. A similar phenomenon has been observed in a recent report on molybdenum-doped BiVO_4_ photoanode^23^. The previous discussion on the XPS data has suggested that phosphorus incorporation alleviated the native oxygen vacancies existed in the BiVO_4_ lattice. Therefore, apart from the substitution of V by phosphorus atoms, the suppressed oxygen vacancies could also contribute to moderating the polaron hopping barrier^17^.

The self-trapping energy of polaron states (the stabilization energy gained upon lattice distortion and localization, Δ*E*_ST_) is calculated as the energy difference between the delocalized and polaronic states^44^. The Δ*E*_ST_ of pristine and phosphorus-doped BiVO_4_ samples were determined to be 0.39 and 0.34 eV (averaged over all polaronic positions considered in Fig. 6a), respectively. The calculated Δ*E*_ST_ values are comparable with the reported values in the literature^13,45^. A schematic diagram of the potential energy landscape for the band states with polaron formation is given in Fig. 6e, the small polaron state of BiVO_4_ becomes less stable and easier to be re-excited to a delocalized band state by photons upon the incorporation of phosphorus, which could facilitate charge transport and reduce the chance of recombination with photoinduced holes as electron transport in band state is more effective than that through localized states.

### PEC water-splitting activity

**Fig. 7:**
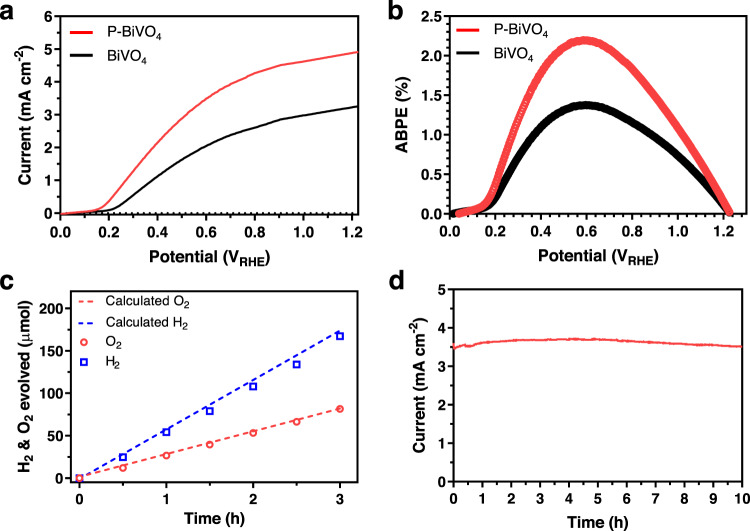
Photoelectrochemical performance for water-splitting. **a** Current density-voltage curves of the BiVO_4_ and phosphorus-doped BiVO_4_ samples with co-catalyst of NiFeO_*x*_ measured under back illumination with AM 1.5 G simulated sunlight for water-splitting. **b** ABPEs of the BiVO_4_ and phosphorus-doped BiVO_4_ samples with NiFeO_*x*_ measured in a three-electrode configuration. **c** Gases evolved from the phosphorus doped BiVO_4_ samples at 0.6 V_RHE_ under back illumination for 3 h and the calculated amounts of gas assuming 100% Faradaic efficiency (dashed line). **d** Chronoamperometry curve of the phosphorus-doped BiVO_4_ sample with co-catalyst of NiFeO_*x*_ measured at 0.6 V_RHE_ under AM1.5 G simulated sunlight.

The PEC water splitting performance of phosphorus-doped BiVO_4_ samples coated with oxidation cocatalyst was tested without a hole scavenger. A NiFeO_*x*_ cocatalyst for the oxygen evolution reaction (OER) was deposited on the phosphorus-doped BiVO_4_ surfaces by pulsed photo-electrodeposition method (details in methods). The *J–V* results show that the phosphorus-doped BiVO_4_ sample coated with NiFeO_*x*_ cocatalyst attains 3.51 ($\pm$0.15) mA cm^−2^ at 0.6 V_RHE_ under AM 1.5 G illumination, which is much higher than that of the bare phosphorus-doped BiVO_4_ and 69% higher than that of the BiVO_4_ sample with NiFeO_*x*_ cocatalyst (Fig. 7a). Moreover, the NiFeO_*x*_/phosphorus-doped BiVO_4_ shows a negatively shifted onset potential of ~320 mV compared to the phosphorus-doped BiVO_4_ sample, which is due to the effectively suppressed surface recombination^46^. Figure 7b shows the half-cell applied bias photon-to-current efficiencies (ABPEs) of the pristine and phosphorus-doped BiVO_4_ samples coated with NiFeO_x_ cocatalyst. The ABPE is calculated to be 2.21% at 0.6 V_RHE_ for the phosphorus-doped BiVO_4_, which is not only higher than the pristine counterpart (1.40% at 0.6 V_RHE_) but also among the highest reported for BiVO_4_ (Supplementary Table 2).

As demonstrated in the video (Supplementary Movie 1), the NiFeO_x_/phosphorus-doped BiVO_4_ sample exhibited a robust water oxidation process at 0.6 V_RHE_ under AM 1.5 G irradiation. The evolved H_2_ and O_2_ gases were measured and compared with the theoretical values shown in Fig. 7c. The ratio of H_2_/O_2_ detected was ~2:1 with a calculated Faradaic efficiency of 98% for O_2_ production at 0.6 V_RHE_. The chronoamperometry result collected at 0.6 V_RHE_ does not show obvious current decay over 10 h, which indicates that the phosphorus-doped BiVO_4_ sample coated with NiFeO_x_ cocatalyst is relatively stable under the operation conditions (Fig. 7d).

## Discussion

**Fig. 8:**
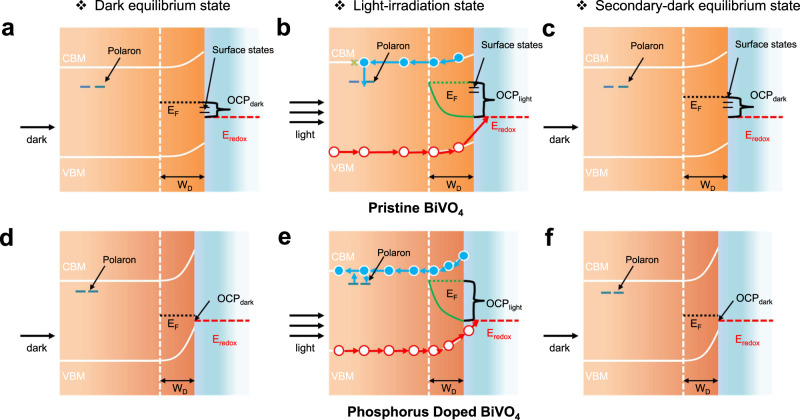
Band structures and band bending schematics of the BiVO4 and phosphorus-doped BiVO4 samples. Band structures and band bending schematics of the BiVO_4_ and phosphorus-doped BiVO_4_ samples. **a, d** Dark equilibrium state, **b, e** light-irradiation state, and **c, f** secondary-dark equilibrium state. The relative positions of conduction band minimum (CBM), valence band maximum (VBM), OCP in the dark (OCP_dark_), OCP under illumination (OCP_light_), depletion width (W_D_), deep defects (solid purple remark beneath CBM) and polaron states (solid blue remark beneath CBM) are constructed from the UV–vis, Mott-Schottky, TRMC, OCP and cNEB simulation data. Adapted and reprinted with permission from Ye, K.-H. et al. Enhancing photoelectrochemical water splitting by combining work function tuning and heterojunction engineering. Nat. Commun. 10, 3687 (2019). Published under (CC-BY) license.

Considering that the poor charge carrier transport of BiVO_4_ photoanode is mainly due to the electron trapping and fast charge recombination, the reduction of polaron hopping barrier and surface trap states frees the self-trapped charge carriers and facilitates the charge transport in BiVO_4_. A schematic diagram of the band structures for the pristine and the phosphorus-doped BiVO_4_ samples is provided in Fig. 8a–f. With increased *N*_D_ and positively shifted *E*_FB_, phosphorus doping steepened the band bending for charge collection at SEI (Fig. 8d–f). Moreover, the cNEB simulation results suggest that *E*_a_ and *E*_ST_ were reduced upon phosphorus doping, moving the polaron state of BiVO_4_ closer to the band edge (Fig. 8b, e). Surface states intrinsically exist in the pristine BiVO_4_ and elevate the Femi-level position in the dark (Fig. 8a). Together with the stronger polaron formation, the surface states reduce the maximum quasi-Fermi-level splitting that can be achieved under light illumination (Fig. 8b), thus resulting in a low V_ph_^13,47^. Benefiting from the passivated surface states, the phosphorus-doped BiVO_4_ sample shows a closer position of Fermi-level (OCP_dark_) relative to the valence band maximum (VBM) in the dark, as shown in Fig. 8d. A similar phenomenon was recently reported for the molybdenum doped BiVO_4_^14^. Under AM 1.5 G irradiation, the Fermi-level position (OCP_light_), CBM and VBM are all elevated, leading to the band flattening in both samples (Fig. 8b, e). At the same time, the quasi Fermi-level splitting (V_ph_) of BiVO_4_ photoanode is enlarged by phosphorus doping, which arises from the truncation of surface states and Fermi-level pinning (Fig. 8e). After turning off the light, the slow communication of the band edges with the deep surface states in the pristine BiVO_4_ slows down the relaxation of OCP_light_ to OCP_dark_ (Fig. 8c). On the other hand, the doped sample relaxed much faster back to the initial OCP_dark_ (Fig. 8f), indicating a reduced number of deep trap states at the surface. Based on the above discussions, we again suggest that phosphorus-doped BiVO_4_ samples have unique properties including efficient light absorption, superior conductivity (carrier density and electron mobility), steeper band bending for hole collection, and enlarged photovoltage. These characteristics promote charge separation, transport, and collection and finally enable considerable charge carriers to travel long distances at low-bias voltage.

In summary, this work has attempted to fundamentally understand the multiple roles of substitutional phosphorus incorporation in mediating charge transport of the nanoporous BiVO_4_ samples. The synergistic effects have allowed the phosphorus-doped BiVO_4_ to exhibit record high photon-to-current conversion efficiencies with APCE of 87% and *η*_sep_ of 80% at 0.6 V_RHE_. NiFeO_*x*_ water oxidation catalyst was coated to facilitate charge injection at the phosphorus-doped BiVO_4_ surface, demonstrating a high ABPE of 2.21%. This study extends the understanding of the doping effect of metal oxide photoelectrodes beyond the increased carrier density to now include charge transport dynamics, particularly with mediating trap states and polaron hopping.

## Methods

### Synthesis of BiVO_4_ and phosphorus-doped BiVO_4_ samples

The BiVO_4_ sample was fabricated following the typical procedure reported in the literature^4^. In brief, BiOI nanoflakes were firstly electrodeposited on the pre-cleaned FTO substrate from a 0.04 M Bi(NO_3_)_3_ solution containing 0.4 M KI and 0.23 M *p*-benzoquinone for 5 mins. Subsequently, 150 µL of DMSO solution containing 0.2 M vanadyl acetylacetonate was impregnated on the prepared BiOI sample at 60 ^o^C and then calcined in Air at 450 ^o^C for 2 h with a ramping rate of 2 ^o^C min^−1^. The obtained samples were soaked in 1 M NaOH solution for 30 mins with gentle stirring to remove the impurities. Lastly, the samples were sequentially rinsed with water and ethanol. Phosphorus-doped BiVO_4_ samples were prepared by a gas phase implementation method. In a typical procedure, 0.5 mg, 1 mg, or 5 mg of NaH_2_PO_2_·H_2_O was placed in the middle of a furnace tube and the fabricated BiVO_4_ sample was located at the downstream side with a distance of 26 cm. Ar flow was purged in the furnace tube for 30 min before elevating the temperature. Subsequently, the temperature of the furnace tube was elevated with a ramping rate of 2 ^o^C min^−1^ and was held at 300 ^o^C for 30 mins under Ar atmosphere. Thermal decomposition of NaH_2_PO_2_·H_2_O led to the formation of phosphine gas. The phosphorus-doped BiVO_4_ samples were carefully taken out from the furnace tube after the temperature naturally cooled down to room temperature. The corresponding treated samples are denoted as P-BiVO_4_−0.5 mg, P-BiVO_4_−1 mg, and P-BiVO_4_−5 mg, respectively.

### Depositing of NiFeO_*x*_ cocatalysts on phosphorus-doped BiVO_4_ samples

NiFeO_x_ water oxidation cocatalyst was deposited on the photoactive surfaces of BiVO_4_ samples by a pulsed photoelectrodeposition method reported^14^. In brief, NiFeO_*x*_ catalyst was deposited on the prepared BiVO_4_ samples at 0 V_RHE_ in 0.4 mM Fe(CH_3_COO)_2_ and 0.04 mM Ni(CH_3_COO)_2_ solution. The backside of the BiVO_4_ sample was irradiated by AM 1.5 G light source with a chopped on/off cycle of 10 s/20 s for 20 min.

### Characterization

Microscopic morphologies were characterized by a scanning electron microscope (FEI Nova Nano SEM 450). The crystal structure of the prepared samples was studied by a PANalytical Empyrean II X-Ray diffractometer (XRD) with a Cu Kα radiation source (λ=1.54060 Å). X-ray photoelectron spectroscopy (XPS) measurements were done using a Thermo Scientific (ESCALAB220i-XL) instrument with a monochromatic Al Kα X-ray source at 1486.68 eV. The presented XPS data and binding energies were calibrated by the carbon 1 *s* peak at 284.6 eV. The high-resolution transmission electron microscopy (HRTEM) images and corresponding energy-dispersive X-ray spectroscopy (EDX) mapping results were obtained using FEI Talos FE200x G2 instrument. The optical absorption properties of the samples were recorded using a UV–Vis–NIR spectrophotometer equipped with an integrating sphere (UV-3600, Shimadzu).

### PEC measurements

PEC measurements were carried out using a CHI 660E potentiostat. A three-electrode cell with a flat quartz window was used, in which a Pt wire and an Ag/AgCl electrode were used as the counter and the reference electrodes, respectively. The electrolyte was 0.1 M KPi buffer solution (pH $=$7). For sulfite oxidation studies, 1 M Na_2_SO_3_ was added to the electrolyte. The electrolytes were purged with argon gas for 30 min before measurement to remove any dissolved oxygen. While the simulated solar irradiance was acquired by illuminating light from a 300 W Xe arc lamp (Oriel Newport) through a neutral density filter and an AM 1.5 G filter. The intensity of the light source was calibrated to 100 mW cm^−2^ at the surface of the FTO side by a Newport optical power meter. The output spectral distribution of the Oriel Newport lamp is calibrated with the standard AM 1.5 G spectrum. *J–V* curves were obtained using linear sweep voltammetry which was performed from 0 to 1.23 V_RHE_ at a scan rate of 10 mV s^−1^. The potential was converted from Ag/AgCl potentials by Eq. (1)

1 $${E}_{{{{{{\rm{RHE}}}}}}}={E}_{{{{{{\rm{Ag}}}}}}/{{{{{\rm{AgCl}}}}}}}+\left(0.059\times {{{{{\rm{pH}}}}}}\right)+0.197.$$

The conductance-frequency curves were determined from EIS results by applying an alternating potential with 5 mV amplitude in the frequency from 1 to 10^5^ Hz at 0.6 V_RHE_. Mott–Schottky results were obtained at a frequency of 10^3^ Hz and a scan rate of 10 mV s^−^^1^ in the dark. The *N*_D_ can be determined using Eq. (2)

2 $${N}_{{{{{{\rm{D}}}}}}}=(2/q\varepsilon {\varepsilon }_{0}){[d(1/{c}^{2})/dV]}^{-1},$$

where *q* is the electron charge, $\varepsilon$ is the dielectric constant of BiVO_4_ semiconductor, ${\varepsilon }_{0}$ is the permittivity of vacuum, *c* is the capacitance of the depletion region, V is the applied voltage, and $d(1/{c}^{2})/dV$ is the slope of the Mott–Schottky plot. The *W*_D_ for materials sharing similar morphology is calculated using Eq. (3)

3 $${W}_{{{{{{\rm{D}}}}}}}={\left[2\varepsilon {\varepsilon }_{0}\left({{{{{\rm{V}}}}}}-{E}_{{{{{{\rm{FB}}}}}}}-{kT}/q\right)/q{N}_{{{{{{\rm{D}}}}}}}\right]}^{1/2},$$

where *E*_FB_ is the flat band position. Open circuit potentials were collected in 0.1 M KPi solution with 1 M Na_2_SO_3_ after testing 2800 s in the dark and under AM 1.5 G irradiation. Temperature-dependent EIS measurement was performed on CHI 660E potentiostat equipped with an oil bath and hot-plate. Under the dark condition, conductance-frequency data of BiVO_4_ samples were determined at different temperatures with an applied bias of 0.6 V_RHE_.

IPCE was examined under monochromatic irradiation from a 300 W Xe lamp (Newport Oriel) equipped with a monochromator (Newport Oriel) at 0.6 V_RHE_ in 0.1 M KPi with 1 M Na_2_SO_3_. The intensity of light (P(*λ*)) was measured by a Newport optical power meter connected with a calibrated silicon photodiode detector. The IPCE values were obtained by Eq. (4)

4 $${{{{{\rm{IPCE}}}}}}\left(\%\right)=\left(1240\times I\right)/\left(\lambda \times P\left(\lambda \right)\right)\times 100,$$

where *λ* is the wavelength and *I* is the photocurrent density. APCE data at each wavelength was calculated according to Eq. (5)

5 $${{{{{\rm{APCE}}}}}}\left(\%\right)={{{{{\rm{IPCE}}}}}}\left(\%\right)/{{{{{\rm{LHE}}}}}}.$$

*J*_abs_ is the photon absorption rate presented as the photocurrent density of BiVO_4_ samples, which is obtained according to Eq. (6)

6 $${J}_{{abs}}=q/{hc}\times {\int }_{\lambda }\,\lambda {Flux}\left(\lambda \right){\eta }_{{{{{{\rm{abs}}}}}}}d\lambda,$$

where *h* is the Plank constant, *c* is the light speed, ${\eta }_{{{{{{\rm{abs}}}}}}}$ is the light absorbance of the BiVO_4_ samples. *η*_sep_ was calculated by Eq. (7)

7 $${\eta }_{{{{{{\rm{sep}}}}}}}\approx {J}_{{{{{{\rm{sulfite}}}}}}}/{J}_{{{{{{\rm{abs}}}}}}},$$

where ${J}_{s{{{{{\rm{ulfite}}}}}}}$ is the photocurrent density obtained in sulfite oxidation (assuming the surface charge injection efficiency for sulfite oxidation is nearly 100%). The ABPE values were determined from the *J-V* curves in a three-electrode system using Eq. (8)

8 $${{{{{\rm{ABPE}}}}}}\left(\%\right)={J}_{{{{{{{\rm{H}}}}}}}_{2}{{{{{\rm{O}}}}}}}\times \left(1.23-{{{{{{\rm{V}}}}}}}_{{{{{{\rm{bias}}}}}}}\right)/P\times 100,$$

where$\,{J}_{{{{{{{\rm{H}}}}}}}_{2}{{{{{\rm{O}}}}}}}$ is the photocurrent density measured in water oxidation, *P* is the light intensity of AM 1.5 G (100 mW cm^−2^) and V_bias_ is the applied bias potential versus RHE.

Gas evolution measurements were performed by side illumination on a gas-tight PEC reactor through a quartz window. The reactor was purged with argon for 30 min before the reaction to remove the dissolved oxygen. The reactions were conducted at 0.6 V_RHE_ for 3 h in 0.1 M KPi under simulated solar irradiation (AM 1.5 G, 100 mW cm^−2^). The accumulated charge (C) was recorded by a potentiostat (CHI 660E). The evolved H_2_ and O_2_ gases were monitored by gas chromatography (Shimadzu GC-8A, HayeSep DB column) using a 5 Å molecular sieve column and Ar as the carrier gas. The stability measurement was conducted by chronoamperometry at 0.6 V_RHE_ for 10 h under simulated solar irradiation (AM 1.5 G, 100 mW cm^−2^).

### TRMC measurements

TRMC results were collected using a wavelength-tunable optical parametric oscillator (OPO) coupled to a diode-pumped Q-switched Nd:YAG laser at wavelengths of 410, 450, and 470 nm as the excitation source with a 3 ns pulse width and an X-band (8400–8700 MHz) microwave probe^48,49^. The samples were mounted in a microwave cavity cell. The signal change ($\triangle P/P$) in the microwave cavity cell correlates to the photoinduced variation in the conductance of the BiVO_4_ sample ($\triangle G$) using Eq. (9)

9 $$\triangle P/P\left(t\right)=-K\triangle G\left(t\right),$$

where *K* is the sensitivity factor derived from the resonance characteristics of the cavity and the dielectric properties of the medium. The product of the charge carrier generation yield ($\phi$) and the sum of electron and hole mobilities (*∑µ*) can be obtained according to Eq. (10)

10 $$\phi \sum \mu=\triangle G/{I}_{0}\beta q{F}_{A},$$

where *I*_*0*_ is the incident intensity per pulse, *q* is the elementary charge, $\beta$ is the ratio between the inner broad and narrow dimensions of the waveguide, and *F*_*A*_ is the fraction of incident photons absorbed within the sample. The laser pulse intensities were adjusted using calibrated filters and varied from ~10^13^ to 4.5  × 10^14^ photons cm^−2^ pulse^−1^. The hole diffusion length (*L*_h_) was calculated using Eq. (11) and Eq. (12)

11 $${L}_{h}=\sqrt{{D}_{h}\tau },\,{D}_{h}={\mu }_{{{{{{\rm{h}}}}}}}{kT}/q$$

12 $${\mu }_{{{{{{\rm{h}}}}}}}=0.7{m}_{0}/\left(0.7{m}_{0}+0.9{m}_{0}\right)\times \mu,$$

where $\mu$, ${\mu }_{{{{{{\rm{h}}}}}}}$, *m*_*0*_, *q*, *T*, and *k* are the carrier mobility, the hole mobility, the effective mass, the electron charge, the temperature, and the Boltzmann constant, respectively^48^.

### Computational details

All density functional theory (DFT) calculations were carried out by Vienna Ab-initio Simulation Package (VASP)^50^. The Perdew-Burke-Ernzerhof (PBE)^51^ type of generalized gradient approximation (GGA)^52^ was applied for exchange-correlation. The electron-core interactions were described by the projector augmented wave (PAW) method^53^. The van der Waals (vdW) interaction was treated by the Grimme method^54^. The plane-wave cutoff energy was set to 400 eV, and the first Brillouin zone was sampled by a 2 × 3 × 3 Monkhorst-pack k-points grid^55^. The effective Hubbard U of 2.7 eV was added to the d orbitals of V atoms according to Dudarev’s method^56^. The structures were fully optimized until the total energy and residual forces were converged to 10^−6^ eV and 0.005 eV Å^−1^, respectively. The polaron hopping was simulated by cNEB method^57,58^ with 7 images, where the first and last images correspond to the initial and final positions of the polaron hopping, respectively.

The formation energy (${E}_{{{{{{\rm{f}}}}}}}$) of phosphorus doping defects including phosphorus interstitial (P_int_), phosphorus on V site (P_V_), phosphorus on Bi site (P_Bi_), and phosphorus on O site (P_O_) was defined as Eq. (13)

13 $${E}_{f}={E}_{{tot}}\left[{BVO},\,{doped}\right]-{E}_{{tot}}\left[{BVO},\,{pristine}\right]-\sum {n}_{i}({{E}_{i}+\mu }_{i})+q\left({E}_{v}+{E}_{{Fermi}}\right)+{E}_{{co}{rr}},$$

where ${E}_{{tot}}\left[{BVO},\,{pristine}\right]$ is the total energy of a perfect supercell (2×1×2 unit cells with 96 atoms) and ${E}_{{tot}}\left[{BVO},\,{doped}\right]$ is the total energy of a supercell doped with one phosphorus atom in charge state *q*. ${n}_{i}$ is the number of atoms (in our case is 1) that were removed (${n}_{i}$ < 0) or added (${n}_{i}$ > 0) to the supercell.$\,{E}_{i}$ and ${\mu }_{i}$ are the average energy of the elemental phase and the chemical potential, respectively. ${E}_{V}$ represents the energy at the valence band maximum of the defect free structure. ${E}_{{Fermi}}$ is the Fermi energy relative to the ${E}_{V}$, ranging from 0 to 2.1 eV (band gap of pristine BVO). The final term$\,{E}_{{corr}}$ represents the correction term for the electrostatic finite-size effect, following the scheme proposed by Freysoldt^59^. The 0, +1, +2, +3, +4, and +5 charge states were considered for P_int_; the 0, +1, and +2 charge states were considered for P_Bi_; the 0 and −1 charge states were considered for P_O_. Only the neutral state of P_V_ was considered because P possesses the same number of valence electrons as V. To maintain thermal equilibrium, the ${\mu }_{{Bi}}$, ${\mu }_{V}$ and ${\mu }_{O}$ should satisfy the Eq. (14)

14 $${\mu }_{{Bi}}+{\mu }_{V}+4{\mu }_{O}={H}^{f}\left[{{{{{\rm{BVO}}}}}}\right],$$

where ${H}^{f}\left[{{{{{\rm{BVO}}}}}}\right]$ is the formation enthalpy of BiVO_4_. The ${\mu }_{i}$ should also be smaller than zero so that the source elements would not aggregate to the elemental phases. To avoid the formation of secondary phases (Bi_2_O_3_, VO_2_, and V_2_O_5_), the ${\mu }_{{Bi}}$, ${\mu }_{V}$ and ${\mu }_{O}$ shall meet Eqs. (15–17)

15 $${2\mu }_{{Bi}}+3{\mu }_{O} < {H}^{f}\left[{{{{{{\rm{Bi}}}}}}}_{2}{{{{{{\rm{O}}}}}}}_{3}\right],$$

16 $${\mu }_{V}+2{\mu }_{O} < {H}^{f}\left[{{{{{\rm{V}}}}}}{{{{{{\rm{O}}}}}}}_{2}\right],$$

17 $${2\mu }_{V}+5{\mu }_{O} < {H}^{f}\left[{{{{{{\rm{V}}}}}}}_{2}{{{{{{\rm{O}}}}}}}_{5}\right],$$

where ${H}^{f}\left[{{{{{{\rm{Bi}}}}}}}_{2}{{{{{{\rm{O}}}}}}}_{3}\right]$, ${H}^{f}\left[{{{{{\rm{V}}}}}}{{{{{{\rm{O}}}}}}}_{2}\right]$, and ${H}^{f}\left[{{{{{{\rm{V}}}}}}}_{2}{{{{{{\rm{O}}}}}}}_{5}\right]$ are the formation enthalpy of Bi_2_O_3_, VO_2_, and V_2_O_5_, respectively. Considering all these constraints, the available range of ${\mu }_{{Bi}}$, ${\mu }_{V}$ and ${\mu }_{O}$ is limited to the grey area in Supplementary Fig. 10. In our calculations, two representative conditions shown as A (O rich) and B (O poor) were considered. The chemical potential of phosphorus element is constrained by ${\mu }_{P}$ <0 and ${2\mu }_{P}+5{\mu }_{O} < {H}^{f}\left[{{{{{{\rm{P}}}}}}}_{2}{{{{{{\rm{O}}}}}}}_{5}\right]$.

### Reporting summary

Further information on research design is available in the Nature Research Reporting Summary linked to this article.

## Supplementary information

Supplementary Information

Description of Additional Supplementary Files

Supplementary Movie 1

Reporting Summary

## Data Availability

The data that support the findings of this study are available from the corresponding author upon reasonable request. Source data are provided as a Source Data file. Source data are provided with this paper.

## Source data

Source Data
