# Supplementary material for: Low-bias photoelectrochemical water splitting via mediating trap states and small polaron hopping
Source: Nat Commun. 2022 Oct 20;13:6231. doi: 10.1038/s41467-022-33905-6 (PMC9585101; doi:10.1038/s41467-022-33905-6)
Supplement: Supplementary file 1 — Supplementary Information [file 41467_2022_33905_MOESM1_ESM.pdf]

Supplementary Information

Low-bias photoelectrochemical water splitting via mediating trap states and small polaron hopping

Hao Wu[1,2], Lei Zhang[3], Aijun Du[3], Rowshanak Irani[4], Roel van de Krol[4], Fatwa. F. Abdi[4], Yun Hau Ng[1,2*]

[1]Low-Carbon and Climate Impact Research Centre, School of Energy and Environment, City University of Hong Kong, 83 Tat Chee Avenue, Kowloon, Hong Kong, SAR. [2]City University of Hong Kong Shenzhen Research Institute, Shenzhen Hi-Tech Industrial Park, Nanshan District, Shenzhen, China. [3]School of Chemistry, Physics and Mechanical Engineering, Queensland University of Technology, Gardens Point Campus, Brisbane, QLD 4001, Australia. [4]Institute for Solar Fuels, Helmholtz-Zentrum Berlin für Materialien und Energie GmbH, Hahn-Meitner-Platz 1, Berlin 14109, Germany.

*Corresponding author: Yun Hau Ng - yunhau.ng@cityu.edu.hk

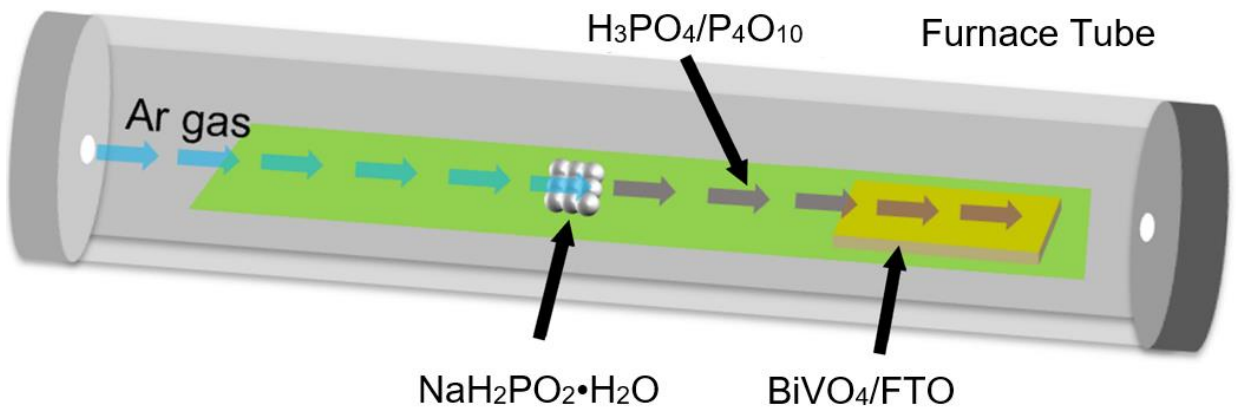

Supplementary Figure 1. Schematic diagram for the preparation of phosphorus-doped $BiVO_4$ sample. Adapted and reprinted with permission from Hu, Z., Shen, Z. & Yu, J. C. Covalent fixation of surface oxygen atoms on hematite photoanode for enhanced water oxidation. Chem. Mater. 28, 564–572 (2016). Copyright 2016 American Chemical Society.

Phosphorus doping in $BiVO_4$ is achieved by exposure to locally generated phosphine gas and water vapor in a tube furnace. With the temperature elevation, the $NaH_2PO_2\cdot H_2O$ precursor is decomposed into phosphine gas and water vapor. The phosphine gas is a strong reducing agent which can likely reduce the evolved water vapor being self-oxidized into H_3PO_4 or P_4O_{10}, and is doped into the oxide lattice structure with an oxidation state of +5 as reported.[1,2] We note that the phosphorus species being transported are likely in the form of H_3PO_4/P_4O_{10}.

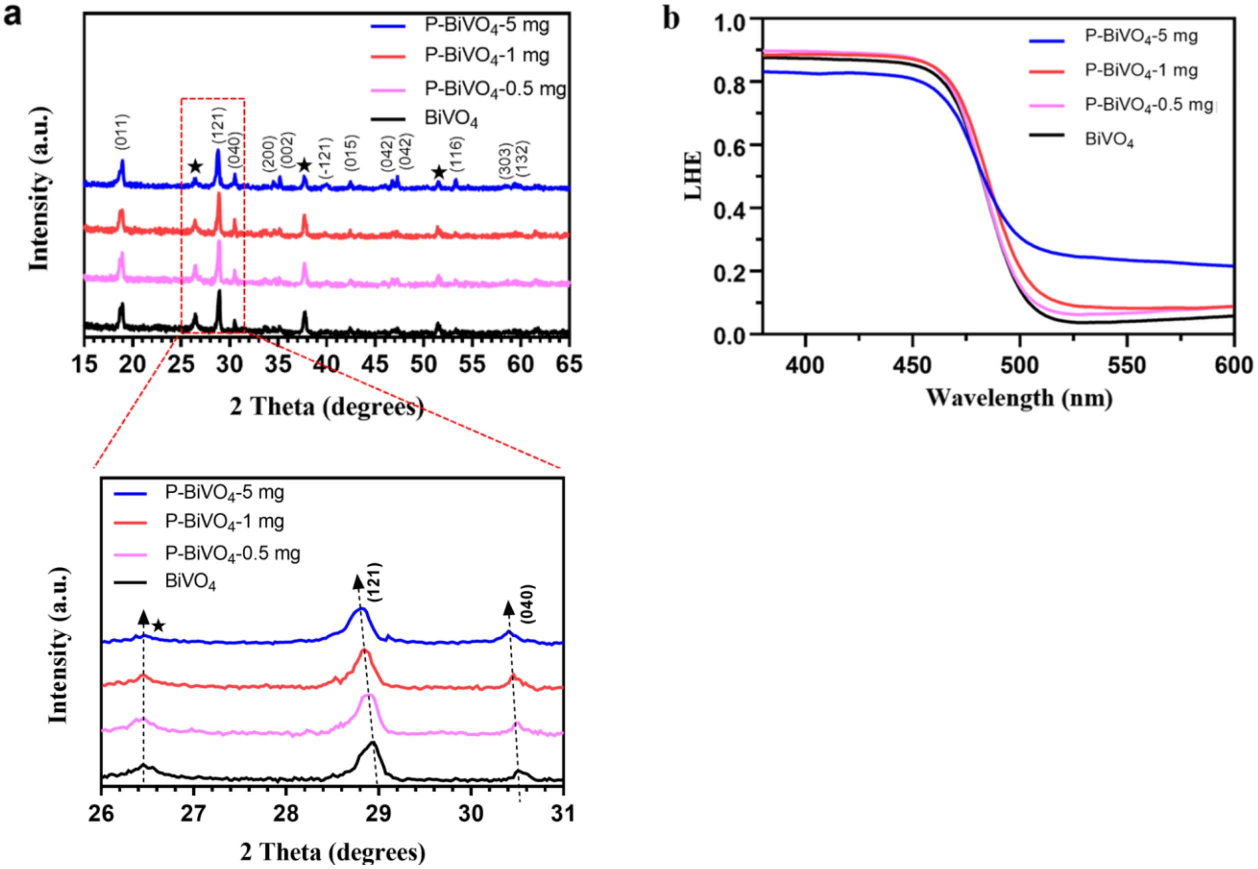

Supplementary Figure 2. Crystallographic and optical characterizations. **a** XRD patterns and **b** LHEs derived from the UV-vis absorption spectra of $BiVO_4$, P-$BiVO_4$-0.5 mg, P-$BiVO_4$-1 mg, and P-$BiVO_4$-5 mg samples. The asterisk remark stands for the intensity peak of the FTO substrate.

The scanning electron microscopy (SEM) images show that both the pristine and phosphorus-doped $BiVO_4$ samples share a similar porous morphology with an average particle size of 80 $\pm$ 3 nm, which is comparable to the reported value in the literature[3]. The crystal structure of the pristine and phosphorus-doped $BiVO_4$ samples was measured by X-ray diffraction (XRD). The XRD patterns of both samples matched the reference pattern for clinobisvanite $BiVO_4$ (generally regarded as monoclinic phase, PDF no. 98-010-0605) and cassiterite SnO_2 (PDF no. 98-003-9178). The magnified XRD patterns of (121) and (040) diffraction peaks show gradual shifts of peak positions toward lower 2 Theta degrees with increasing the phosphorus precursor amount, suggesting the increased strain of the $BiVO_4$ upon the incorporation of phosphorus. Besides, the DFT calculation results also show that the simulated P-O and V-O bond distances are 1.559 Å and 1.742 Å, respectively, indicating the reduced grain size of the phosphorus-doped $BiVO_4$ photoanodes. Therefore, the observed shifts of the diffraction peaks are consistent with the DFT calculations.

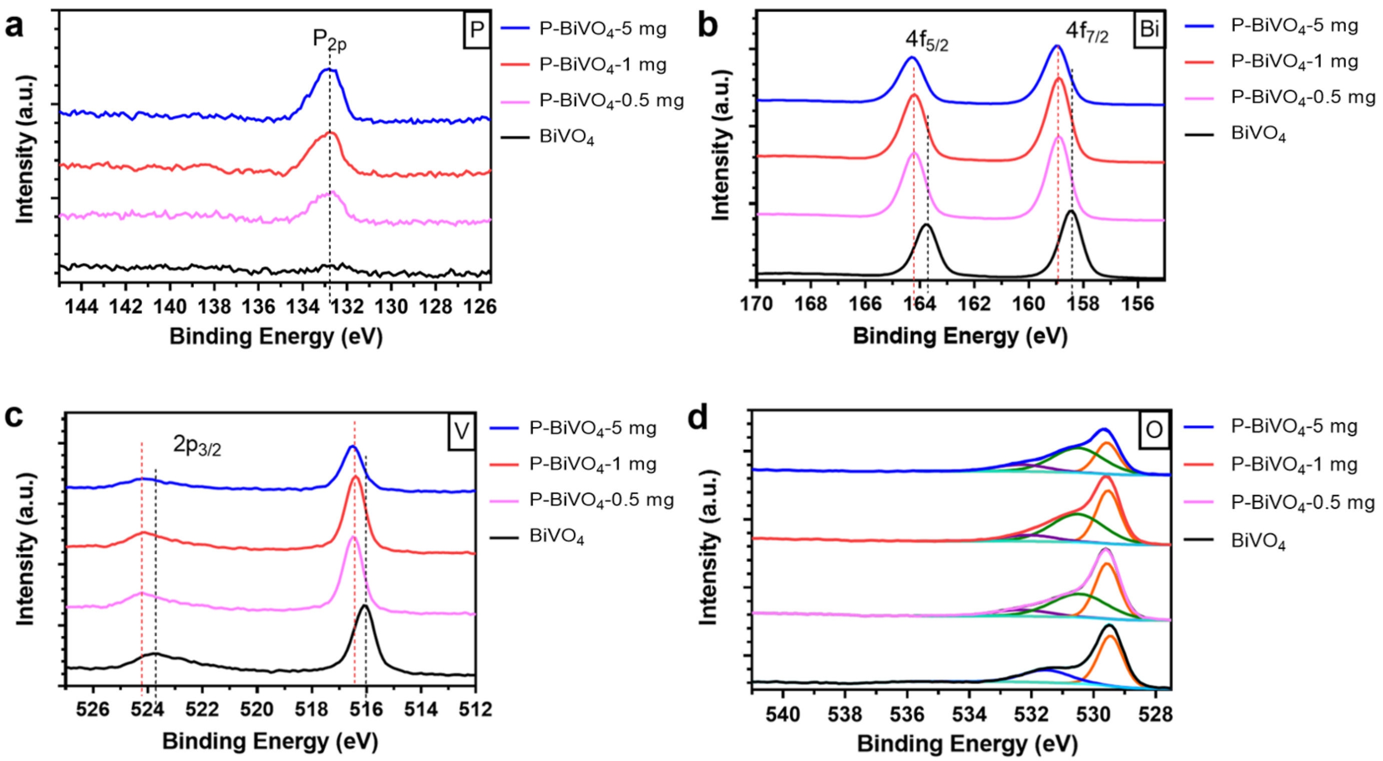

Supplementary Figure 3. Electronic structure characterizations. **a** P 2*p*, **b** Bi 4*f*, **c** V 2*p*, and **d** O 1*s* XPS of the $BiVO_4$, P-$BiVO_4$-0.5 mg, P-$BiVO_4$-1 mg, and P-$BiVO_4$-5 mg samples.

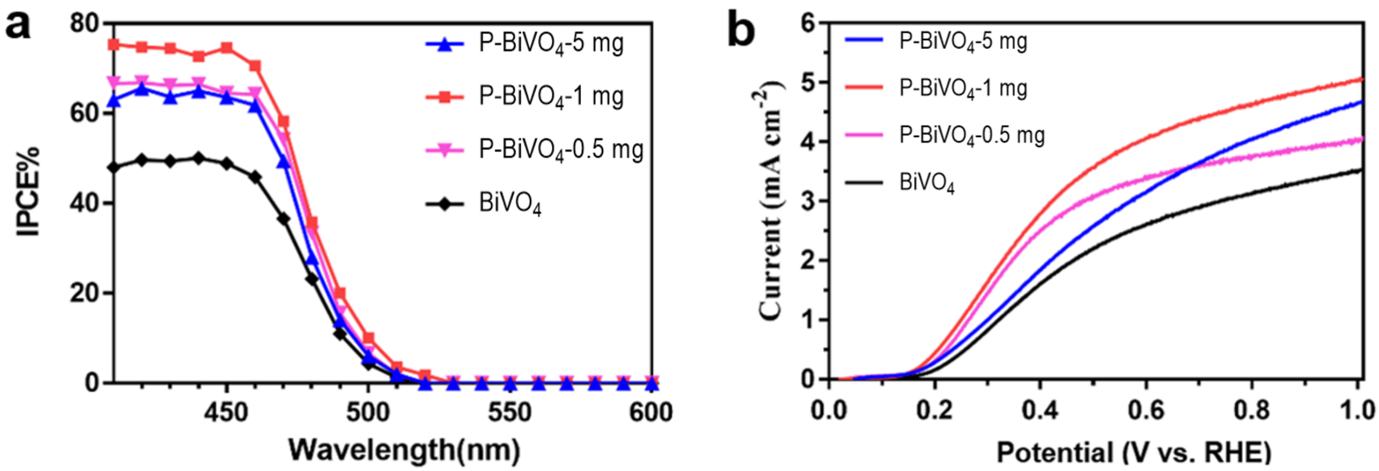

Supplementary Figure 4. Photoelectrochemical performance for sulfite oxidation. **a** IPCE curves of the $BiVO_4$, P-$BiVO_4$-0.5 mg, P-$BiVO_4$-1 mg and P-$BiVO_4$-5 mg samples measured at 0.6 V_{RHE}. **b** Current density-voltage curves of the $BiVO_4$, P-$BiVO_4$-0.5 mg, P-$BiVO_4$-1 mg and P-$BiVO_4$-5 mg samples measured under back illumination with AM 1.5G simulated sunlight.

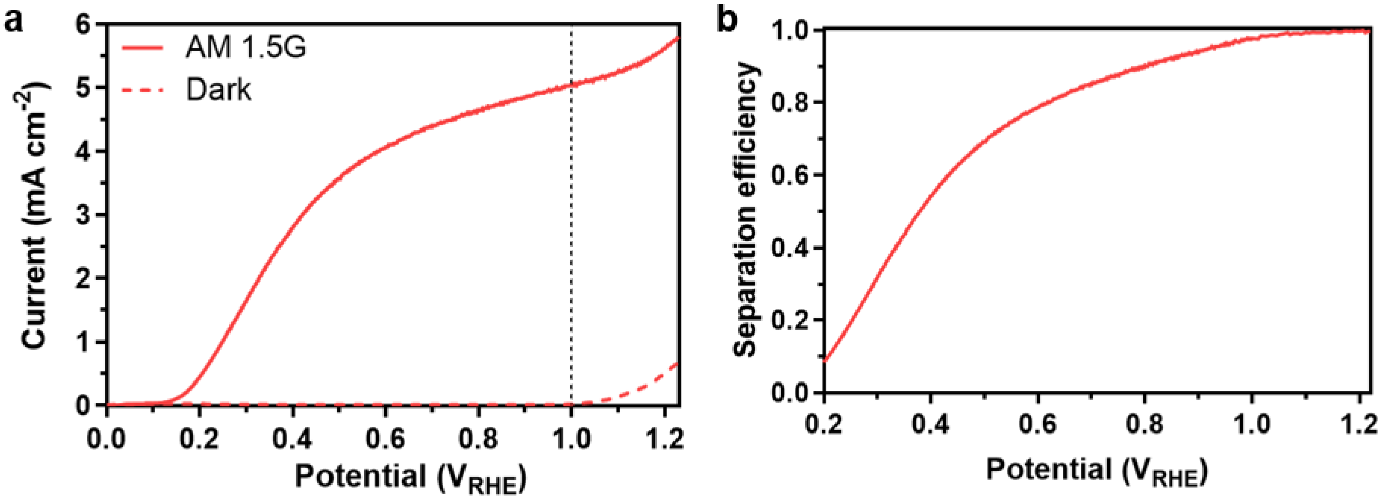

Supplementary Figure 5. Photoelectrochemical performance for sulfite oxidation. **a** *J*-*V* curves of the phosphorus-doped $BiVO_4$ photoanode under the AM 1.5G illumination and in the dark in the electrolyte containing SO_3^{2-}. **b** η_{sep} calculated from the net photocurrent density-voltage curves for the phosphorus-doped $BiVO_4$ photoanode.

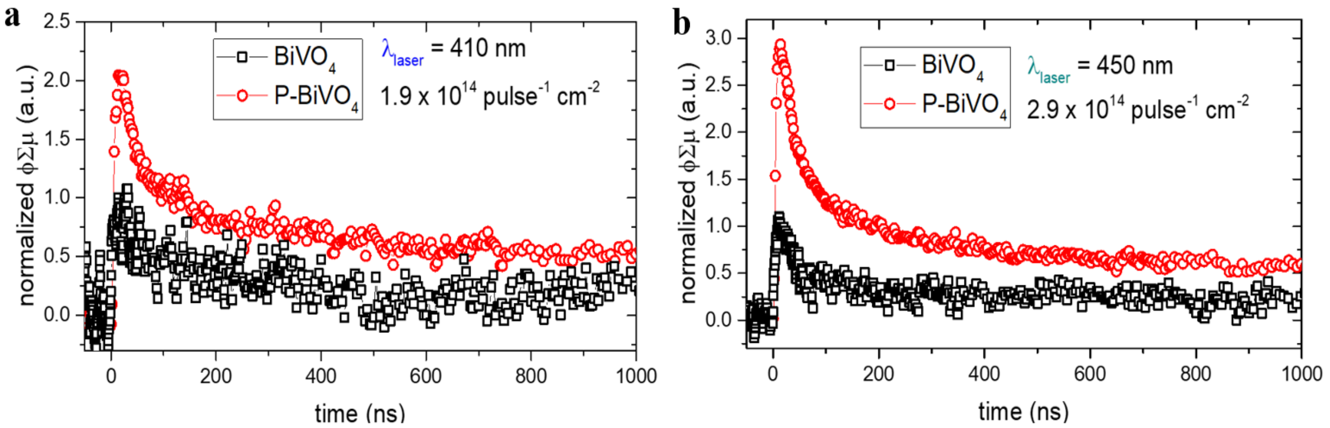

Supplementary Figure 6. Charge transport dynamics of the $BiVO_4$ and phosphorus-doped $BiVO_4$ samples. TRMC results for the pristine $BiVO_4$ and P-BVO_4-1 mg samples measured under pulsed laser light at **a** 410 nm with intensity of 1.9×10^{14} photons pulse^{-1} cm^{-2} and **b** 450 nm with intensity of 2.9×10^{14} photons pulse^{-1} cm^{-2}, respectively.

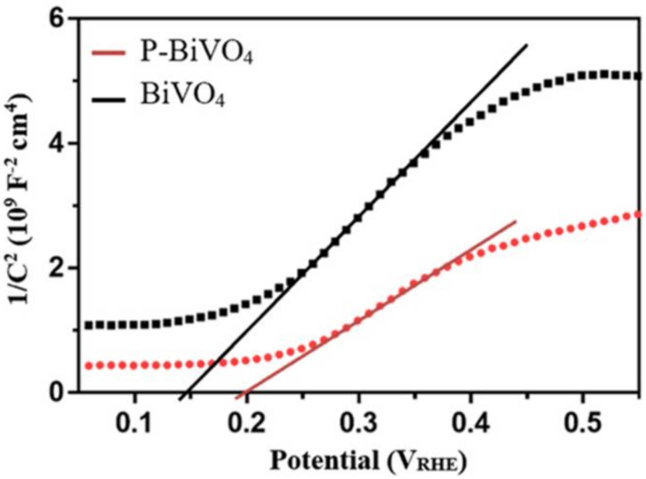

Supplementary Figure 7. Photoelectrochemical measurement. Mott-Schottky plots for the pristine $BiVO_4$ and P-$BiVO_4$-1 mg measured under AM 1.5G simulated sunlight.

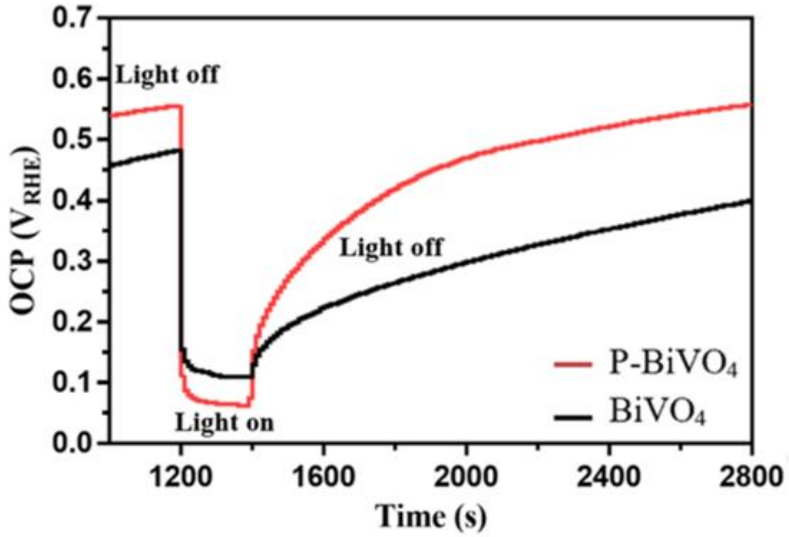

Supplementary Figure 8. Photoelectrochemical measurement. Transient OCP profiles of the $BiVO_4$ and P-$BiVO_4$-1 mg samples.

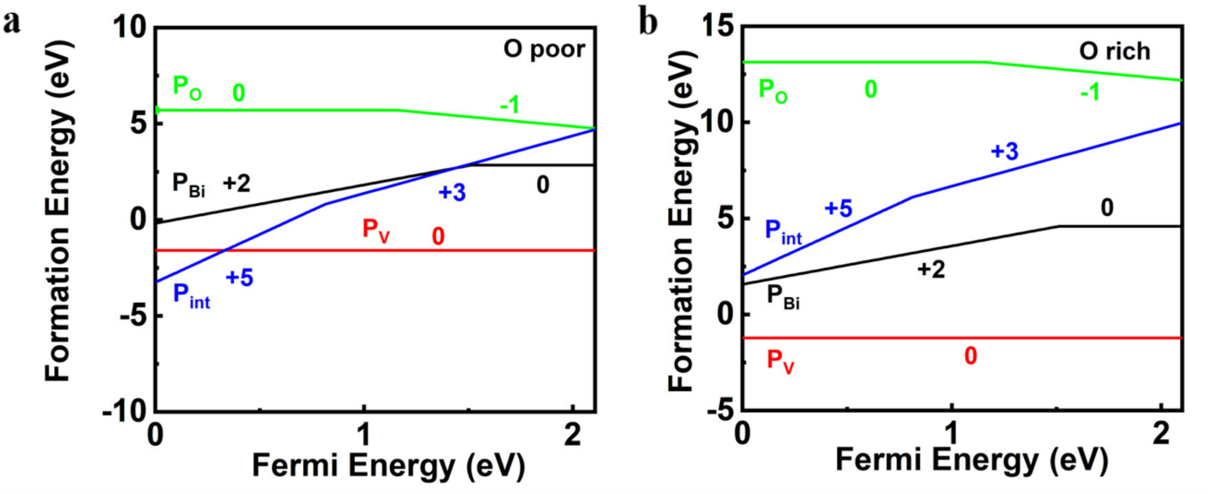

Supplementary Figure 9. DFT calculation. The formation energy of phosphorus defect as a function of Fermi energy under **a** the O-poor and **b** the O-rich conditions. Only the charge states with the lowest formation energy are shown for each type of defect.

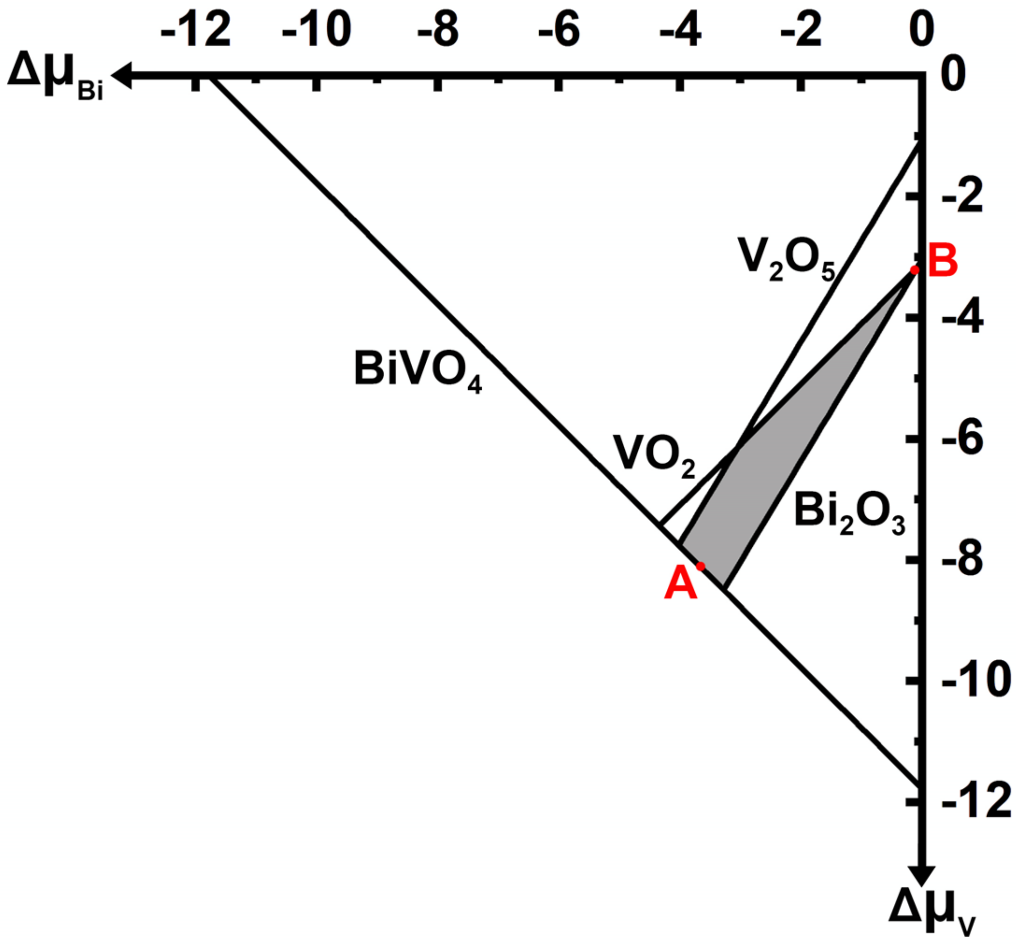

Supplementary Figure 10. DFT calculation. The available range of chemical potentials for equilibrium growth of $BiVO_4$ (grey area). A and B represent the O rich and O poor conditions respectively.

Supplementary Table 1. The performance of state-of-the-art $BiVO_4$ photoanodes for low-bias PEC sulfite oxidation.

Photoanode		Modification	Current ($mA\ cm^{-2}$) @$0.6V_{RHE}$	IPCE% @$0.6V_{RHE}$-410 nm	APCE% @$0.6V_{RHE}$-410 nm	η_{sep}% @$0.6V_{RHE}$	Ref
**Doped $BiVO_4$**	**P doped $BiVO_4$**	**P doping**	**4.08**	**75**	**87**	**80.0**	**This work**
	b-$BiVO_4$	H doping	≈4.0	n. a.	n. a.	60	4
	Sm doped $BiVO_4$	Sm doping	3.85	n. a.	n. a.	n. a.	5
	N doped $BiVO_4$	N doping	4.16	62	72	76	6
	W doped $BiVO_4$	W gradient doping	≈2.5	n. a.	n. a.	≈20	7
	Mo doped $BiVO_4$	Mo doping	3.2	n. a.	n. a.	45	8
	Mo doped $BiVO_4$	Mo doping + Nanostructuring + SnO_2 heterojunction	≈3.8	n. a.	n. a.	n. a.	9
	H doped $BiVO_4$	H doping	1.5	36	n. a.	n. a.	10
	S doped $BiVO_4$	S doping	0.6	n. a.	n. a.	n. a.	11
**Hetero-structured $BiVO_4$**	B-CN-Mo: $BiVO_4$	B-CN heterojunction	4.7	≈71	≈79	79	12
	BP/ $BiVO_4$	BP heterojunction	3.9	n. a.	n. a.	75	13
	$BiVO_4$-VO	$V_{13}O_{16}$ heterojunction	n. a.	69[a]	n. a.	n. a.	14
	CQDs/ $BiVO_4$	CQDs heterojunction	4.41	75	82	50	15
	$BiVO_4$/Sb:SnO_2	Sb:SnO_2 heterojunction	4.08	76	n. a.	71.1	16
	Mo doped $BiVO_4$/SnO_2/Si	Mo: BVO/SnO_2/Si heterojunction	3.0	36	n. a.	n. a.	17
	$BiVO_4$/SnO_2	SnO_2 heterojunction	2.8	n. a.	n. a.	n. a.	18
	$BiVO_4$/ WO_3	WO_3 heterojunction	[3.0, 2.0]	n. a.	n. a.	n. a.	19,20
	Multi-layered $BiVO_4$	BVO-BVO heterojunction	2.6	n. a.	n. a.	n. a.	21
**Pristine $BiVO_4$**	$BiVO_4$	Nanostructuring	≈4.0	80	n. a.	n. a.	22
	$BiVO_4$	Nanostructuring	3.3	60	72	70	3
	$BiVO_4$	Crystallographic orientation	3.5	n. a.	n. a.	n. a.	23
	$BiVO_4$	Electrochemical treatment	1.8	n. a.	n. a.	≈29.2	24
	$BiVO_4$	Oxygen vacancy	≈2.5	n. a.	n. a.	50	25
	$BiVO_4$	Oxygen vacancy	≈4.5	n. a.	n. a.	≈75	26

a. The IPCE was measured at 0.7 V_{RHE};

Supplementary Table 2. The ABPE by state-of-the-art $BiVO_4$ photoanodes for low-bias PEC water splitting.

Photoanode		Modification	Co-catalyst	ABPE%	Potential V_{RHE}	Ref
**Doped $BiVO_4$**	**P doped $BiVO_4$**	**P doping**	**$NiFeO_x$**	**2.21**	**0.6**	**This work**
	b-$BiVO_4$	H doping	TiO_{2-x}	2.50	0.58 V_{Pt}	4
	N doped $BiVO_4$	N doping	FeOOH/NiOOH	2.20	0.58 V_{Pt}	6
	Mo doped $BiVO_4$	Mo doping	TANF	1.56	0.72	8
	Mo doped $BiVO_4$	Mo doping + Nanostructuring + SnO_2 heterojunction	Fe(Ni)OOH	2.05	0.62	9
**Hetero-structured $BiVO_4$**	B-CN-Mo: $BiVO_4$	B-CN heterojunction	$NiFeO_x$	2.67	0.54	12
	$BiVO_4$	Co_3O_4 heterojunction	Co_3O_4	≈0.66	0.83	27
	$BiVO_4$-VO	$V_{13}O_{16}$ heterojunction	Co-Pi	1.55	0.7	14
	CQDs/ $BiVO_4$	CQDs heterojunction	FeOOH/NiOOH	2.29	0.6	15
	$BiVO_4$	BiOI heterojunction	n.a.	0.97	0.68	27
	$BiVO_4$/ WO_3	WO_3 heterojunction	FeOOH/NiOOH	1.65	0.72	19
**Pristine $BiVO_4$**	$BiVO_4$	Nanostructuring	$NiFeO_x$-Bi	2.02	0.6	22
	$BiVO_4$	Nanostructuring	FeOOH/NiOOH	1.75	0.6	3
	$BiVO_4$	Nanostructuring	CoO_x/NiO	1.50	≈ 0.67	28
	$BiVO_4$	Electrochemical treatment	Co-Bi	1.1	0.7	24
	$BiVO_4$	Oxygen vacancy	$NiFeO_x$	1.85	0.67	26

Supplementary References

1. Hu, Z., Shen, Z. & Yu, J. C. Covalent Fixation of Surface Oxygen Atoms on Hematite Photoanode for Enhanced Water Oxidation. *Chem. Mater.* **28**, 564–572 (2016).

2. Zhang, Y. *et al.* Nonmetal P-doped hematite photoanode with enhanced electron mobility and high water oxidation activity. *Energy Environ. Sci.* **8**, 1231–1236 (2015).

3. Woo Kim, T. & Choi, K.-S. Nanoporous $BiVO_4$ Photoanodes with Dual-Layer Oxygen Evolution Catalysts for Solar Water Splitting. *Science.* **343**, 990–994 (2014).

4. Tian, Z. *et al.* Novel Black $BiVO_4/TiO_{2-x}$ Photoanode with Enhanced Photon Absorption and Charge Separation for Efficient and Stable Solar Water Splitting. *Adv. Energy Mater.* **9**, 1901287 (2019).

5. Govindaraju, G. V., Morbec, J. M., Galli, G. A. & Choi, K. S. Experimental and computational investigation of lanthanide ion doping on $BiVO_4$ Photoanodes for Solar Water Splitting. *J. Phys. Chem. C* **122**, 19416–19424 (2018).

6. Kim, T. W., Ping, Y., Galli, G. A. & Choi, K. S. Simultaneous enhancements in photon absorption and charge transport of bismuth vanadate photoanodes for solar water splitting. *Nat. Commun.* **6**, 8769 (2015).

7. Abdi, F. F. *et al.* Efficient solar water splitting by enhanced charge separation in a bismuth vanadate-silicon tandem photoelectrode. *Nat. Commun.* **4**, 2195 (2013).

8. Shi, Y. *et al.* Boosting Photoelectrochemical Water Oxidation Activity and Stability of Mo-Doped $BiVO_4$ through the Uniform Assembly Coating of NiFe-Phenolic Networks. *ACS Energy Lett.* **3**, 1648–1654 (2018).

9. Qiu, Y. *et al.* Efficient solar-driven water splitting by nanocone $BiVO_4$-perovskite tandem cells. *Sci. Adv.* **2**, e1501764 (2016).

10. Jang, J.-W. *et al.* Enhancing Charge Carrier Lifetime in Metal Oxide Photoelectrodes through Mild Hydrogen Treatment. *Adv. Energy Mater.* **7**, 1701536 (2017).

11. Lamers, M. *et al.* Enhanced Carrier Transport and Bandgap Reduction in Sulfur-Modified $BiVO_4$ Photoanodes. *Chem. Mater.* **30**, 8630-8638 (2018) .

12. Ye, K.-H. *et al.* Enhancing photoelectrochemical water splitting by combining work function tuning and heterojunction engineering. *Nat. Commun.* **10**, 3687 (2019).

13. Zhang, K. *et al.* Black phosphorene as a hole extraction layer boosting solar water splitting of oxygen evolution catalysts. *Nat. Commun.* **10**, 2001 (2019).

14. Ren, H. *et al.* Manipulation of Charge Transport by Metallic $V_{13}O_{16}$ Decorated on Bismuth Vanadate Photoelectrochemical Catalyst. *Adv. Mater.* **31**, 1807204 (2019).

15. Ye, K.-H. *et al.* Carbon quantum dots as a visible light sensitizer to significantly increase the solar water splitting performance of bismuth vanadate photoanodes. *Energy Environ. Sci* **10**, 772 (2017).

16. Zhou, L. *et al.* High Light Absorption and Charge Separation Efficiency at Low Applied Voltage from Sb-Doped $SnO_2/BiVO_4$ Core/Shell Nanorod-Array Photoanodes. *Nano Lett* **16**, 39 (2016).

17. Zhang, L. *et al.* Significantly enhanced photocurrent for water oxidation in monolithic Mo:$BiVO_4$ /SnO_2/Si by thermally increasing the minority carrier diffusion length Significantly enhanced photocurrent for water oxidation in monolithic Mo:$BiVO_4$/SnO_2/Si by thermall. *Energy Environ. Sci* **9**, 2044 (2016).

18. Byun, S., Kim, B., Jeon, S. & Shin, B. Effects of a SnO_2 hole blocking layer in a $BiVO_4$-based photoanode on photoelectrocatalytic water oxidation. *J. Mater. Chem. A* **5**, 6905–6913 (2017).

19. Zhou, Y. *et al.* Highly Efficient Photoelectrochemical Water Splitting from Hierarchical WO_3/$BiVO_4$ Nanoporous Sphere Arrays. *Nano Lett.* **17**, 8012–8017 (2017).

20. Pihosh, Y. *et al.* Photocatalytic generation of hydrogen by core-shell WO_3/$BiVO_4$ nanorods with ultimate water splitting efficiency. *Sci. Rep.* **5**, 11141 (2015).

21. Wu, J. M. *et al.* Multi-layer monoclinic $BiVO_4$ with oxygen vacancies and V^{4+} species for highly efficient visible-light photoelectrochemical applications. *Appl. Catal. B Environ.* **221**, 187–195 (2018).

22. Kuang, Y. *et al.* A front-illuminated nanostructured transparent $BiVO_4$ photoanode for >2% efficient water splitting. *Adv. Energy Mater.* **6**, 1501645 (2016).

23. Han, H. S. *et al.* Boosting the solar water oxidation performance of a $BiVO_4$ photoanode by crystallographic orientation control. *Energy Environ. Sci.* **11**, 1299–1306 (2018).

24. Wang, S., Chen, P., Yun, J. H., Hu, Y. & Wang, L. An Electrochemically Treated $BiVO_4$ Photoanode for Efficient Photoelectrochemical Water Splitting. *Angew. Chem. Int. Ed.* **56**, 8500–8504 (2017).

25. Qiu, W. *et al.* Freeing the Polarons to Facilitate Charge Transport in $BiVO_4$ from Oxygen Vacancies with an Oxidative 2D Precursor. *Angew. Chem. Int. Ed.* **8**, 19087 –19095 (2019).

26. Wang, S. *et al.* In Situ Formation of Oxygen Vacancies Achieving Near-Complete Charge Separation in Planar $BiVO_4$ Photoanodes. *Adv. Mater.* **32**, 2001385 (2020).

27. Chang, X. *et al.* Enhanced Surface Reaction Kinetics and Charge Separation of p-n Heterojunction Co_3O_4/$BiVO_4$ Photoanodes. *J. Am. Chem. Soc.* **137**, 8356–8359 (2015).

28. Zhong, M. *et al.* Surface modification of CoO_x loaded $BiVO_4$ photoanodes with ultrathin p-type NiO layers for improved solar water oxidation. *J. Am. Chem. Soc.* **137**, 5053–5060 (2015).
