# Supplementary material for: Low-bias photoelectrochemical water splitting via mediating trap states and small polaron hopping
Source: Nat Commun. 2022 Oct 20;13:6231. doi: 10.1038/s41467-022-33905-6 (PMC9585101; doi:10.1038/s41467-022-33905-6)
Supplement: Supplementary file 2 — Description of Additional Supplementary Files [file 41467_2022_33905_MOESM2_ESM.pdf]

Description of Additional Supplementary Files

File Name: Supplementary Movie 1
Description: Oxygen evolution of the $NiFeO_x$/phosphorus-doped $BiVO_4$ sample.
